# Localization of Secondary Metabolites in Marine Invertebrates: Contribution of MALDI MSI for the Study of Saponins in Cuvierian Tubules of *H. forskali*

**DOI:** 10.1371/journal.pone.0013923

**Published:** 2010-11-10

**Authors:** Séverine Van Dyck, Patrick Flammang, Céline Meriaux, David Bonnel, Michel Salzet, Isabelle Fournier, Maxence Wisztorski

**Affiliations:** 1 Laboratoire de Biologie marine, Université de Mons - UMONS, Mons, Belgium; 2 Laboratoire de Neuroimmunologie et Neurochimie Evolutives, Université Lille Nord de France (USTL), Villeneuve d'Ascq, France; University of Giessen Lung Center, Germany

## Abstract

**Background:**

Several species of sea cucumbers of the family Holothuriidae possess a particular mechanical defense system called the Cuvierian tubules (Ct). It is also a chemical defense system as triterpene glycosides (saponins) appear to be particularly concentrated in Ct. In the present study, the precise localization of saponins in the Ct of *Holothuria forskali* is investigated. Classical histochemical labeling using lectin was firstly performed but did not generate any conclusive results. Thus, MALDI mass spectrometry Imaging (MALDI-MSI) was directly applied and completed by statistical multivariate tests. A comparison between the tubules of relaxed and stressed animals was realized.

**Results:**

These analyses allowed the detection of three groups of ions, corresponding to the isomeric saponins of the tubules. Saponins detected at *m/z* 1287 and 1303 were the most abundant and were apparently localized in the connective tissue of the tubules of both relaxed and stressed individuals. Saponins at *m/z* 1125 and 1141 were detected in lower amount and were present in tissues of relaxed animals. Finally, saponin ions at 1433, 1449, 1463 and 1479 were observed in some Ct of stressed holothuroids in the outer part of the connective tissue. The saponin group *m/z* 14xx seems therefore to be stress-specific and could originate from modifications of the saponins with *m/z* of 11xx.

**Conclusions:**

All the results taken together indicate a complex chemical defense mechanism with, for a single organ, different sets of saponins originating from different cell populations and presenting different responses to stress. The present study also reflects that MALDI-MSI is a valuable tool for chemical ecology studies in which specific chemical signalling molecules like allelochemicals or pheromones have to be tracked. This report represents one of the very first studies using these tools to provide a functional and ecological understanding of the role of natural products from marine invertebrates.

## Introduction

Sea cucumbers seem to be vulnerable animals regarding their numerous predators referenced in the literature [1]. However, many authors consider predation on adult holothuroids to be infrequent [2], [3]. Amongst the numerous anti-predation mechanisms developed by these animals, the toxicity of the body wall and the presence of Cuvierian tubules seems to be the most effective against non-specialist predators [4], [5]. Cuvierian tubules are little caeca located in the posterior part of the animal, that can be ejected toward a predator in response to an aggression [6]. Expelled tubules lengthen into sticky white threads susceptible to entangle the predator [7], [8]. Although only some species of holothuroids from the family Holothuriidae, including *Holothuria forskali*, possess Cuvierian tubules, all the sea cucumbers contain saponins in their body wall and viscera. Saponins of sea cucumbers are secondary metabolites having a triterpene glycoside structure. They have long been suggested to play a role in the defense of these animals as a toxin [4], [9]. Indeed, due to their membranolytic action [10], [11], saponins have a wide range of pharmacological effects such as cytotoxicity to tumor cells, or antifungal and hemolytic actions [12], [13]. Triterpene glycosides are particularly concentrated in the Cuvierian tubules of holothuroids [14], [15] and specific congener mixtures are usually associated with the defensive function of this system [12], [16], [17], [18].

Recent studies demonstrated that mass spectrometry (MS) procedures represent very valuable techniques for the detection and identification of saponins [17]–[20]. However, classical techniques require the complete pounding of the organs to allow the extraction of their contents. In consequence, the determination of the precise localization of saponins in the tissues is impossible. For 10 years, MALDI-MSI (Matrix-Assisted Laser Desorption/Ionization-Mass Spectrometry Imaging), also called MALDI-Imaging, has undergone many developments [21], [22] and has achieved a certain maturity, allowing it to be now used in many domains like clinical proteomics [23] or pharmaceutical fields [24]. This procedure permits to detect and localize ions of interest directly on tissue sections and nearly without any preparation. The benefit of this technique is the high sensitivity and the use of a soft ionization technique, like MALDI-MS, which allows a label free molecular imaging of a biological tissue section. The great added value of using MALDI-MS direct tissue analysis is to detect hundred of molecules in one spatially-resolved analysis. By this way, MALDI-MSI or MALDI profiling will open the door for important breakthroughs in the field of chemical ecology.

In the present study, we investigated the triterpene glycosides of the Cuvierian tubules in the sea cucumber *H. forskali* and attempted to localize saponins in their tissues. We combined classical histochemical labelling to MALDI-MS direct tissue profiling and MALDI-MSI in order to detect saponins and describe their spatial localization in the Cuvierian tubules. A statistical multivariate test using the Principal Component Analysis (PCA) method which allows highlighting groups of close ions from dense and complex data sets was conducted to compare the molecular data of Cuvierian tubules from stressed and relaxed holothuroids.

## Results and Discussion

### Use of lectins to localize saponins on tissue sections

As no antibodies to saponins are available, lectins were considered as a tool to detect these molecules on Cuvierian tubule sections. Lectins are proteins or glycoproteins of non-immune origin that are able to bind specific carbohydrate motifs in a way similar to the formation of the antibody-antigen complex [25]. Each lectin recognizes specifically one oligosaccharidic chain but it may also bind to similar oligo- or mono-saccharides, although with a lower affinity. To the best of our knowledge, no commercially available lectin is specific of the saponin carbohydrate moiety which encloses glucose (Glc), 3-*O*-methylglucose (MeGlc), quinovose (Qui) and xylose (Xyl) residues. However, mannose-binding lectins also show an affinity for glucose [25] and could therefore label saponins.

Three lectins specific to glucose- and mannose-containing oligosaccharides (Con A, LCA and PSA) were therefore selected. To confirm that these lectins are able to recognize the saccharidic chain of the saponins of *H. forskali*, we used a lectin-binding assay based on the technique developed by Smith [26] to detect cell surface glycolipids of human and bovine erythrocytes on thin layer chromatograms (TLC). These lectin-binding assays were realized directly on saponin extracts spotted onto nitrocellulose membrane and TLC plate strips. The development of the dark blue staining at the level of the saponin spots varied with the lectin used except for the positive control for which the reaction was always strong and fast (data not show). Although the three lectins labeled the saponin spots, Con A presented the strongest and fastest reaction compared to LCA and PSA which were both characterized by a much lighter staining. These results indicated that the considered lectins probably bind to the saccharidic chain of saponins and thus could also be used on Cuvierian tubule sections.

**Figure 1:**
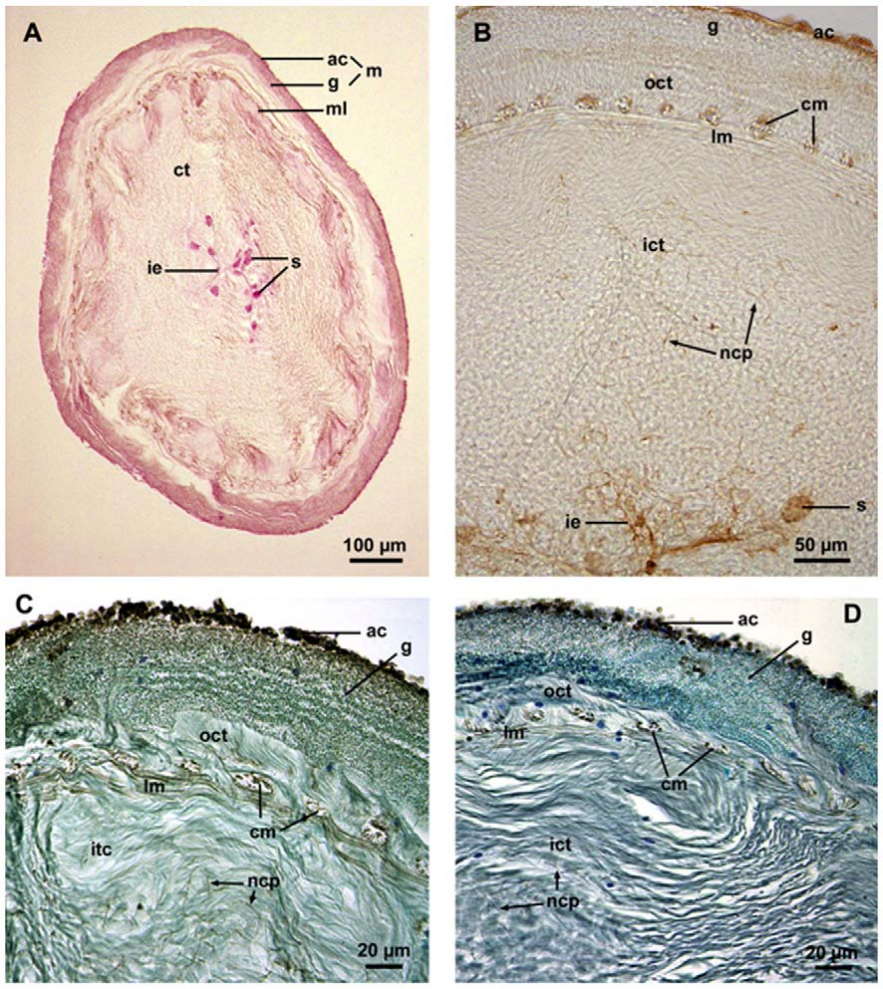
Histochemical labeling of the Cuvierian tubules of *Holothuria forskali.* (A, C, D: paraffin sections; B: cryo-section). A: Cross section through a whole tubule stained with the PAS method; B–D: Transverse sections through the tubule wall labeled with PSA, Con A and LCA, respectively (C and D were counterstained). ac: adluminal cell layer of the mesothelium; cm: circular muscle; ct: connective tissue; g: granular cell layer of the mesothelium; ict: inner connective tissue layer; ie: inner epithelium; lm: longitudinal muscle; m: mesothelium; ml: muscular layer; ncp: neurosecretory-like cell processes; oct: outer connective tissue layer; s: spherulocytes.

Most of the ultrastructural information available on Cuvierian tubules comes from VandenSpiegel and Jangoux [27] and VandenSpiegel et al. [28], who clarified much of the structure and function of these organs in the species *H. forskali*. Quiescent Cuvierian tubules are hollow organs consisting of a narrow central lumen surrounded by a thick wall. The tubule wall is made up of an outer mesothelium and an inner epithelium encompassing a thick connective tissue sheath (Fig. 1A). This sheath includes longitudinal and circular muscle fibers that separate it into a thick, collagen-rich inner layer and a much thinner outer layer. The other cell types present in the connective tissue sheath are the vacuolar cells, always present in the vicinity of the muscular layer, and the neurosecretory-like cells whose processes form an extensive network in the inner connective tissue layer [6]. The mesothelium is the tissue involved in the adhesive process. It is a pseudostratified epithelium made up of two superposed cell layers, an outer layer of adluminal cells and an inner layer of granular cells which is highly folded along the long axis of the tubule. The inner epithelium also consists of two cell types: inner epithelial cells and spherulocytes [6]. Before the lectin experiments, the PAS method was used on Cuvierian tubule sections for staining structures containing a high proportion of carbohydrate-containing molecules and macromolecules (Fig. 1A). PAS-positive molecules are present on the whole section but mainly concentrated in the mesothelium (especially in adluminal cells), in the muscular fibers, and in the inner epithelium (especially in spherulocytes).

The three selected lectins specific (Con A, LCA and PSA) were then used to label the Cuvierian tubules. Investigations were performed on paraffin sections and on cryo-sections. The pattern of labeling was very similar on both types of sections and with the three lectins tested (Figs. 1B–D). The major difference laid in the labeling intensity, reactivity with Con A being always stronger than with the other two lectins (Fig. 1C). In the mesothelium, only adlumnial cells were intensely labeled at the level of large intra-cytoplasmic vesicles. Muscle cells also presented important lectin reactivity. In the inner epithelium, both the inner epithelial cells and the spherulocytes were lightly labeled, the former with Con A and the latter with LCA and PSA (Figs. 1B–D). Finally, two types of lectin reactive structures were also observed in the connective tissue sheath: a few scattered vacuolar cells and the network of narrow cell processes from neurosecretory-like cells.

Although several cell types were reactive with the three lectins tested, this does not mean that all these cells contain saponins. Indeed these three lectins certainly label other molecules like tissue glycoproteins. It was postulated that comparison between paraffin and cryo-sections could give a clue about saponin localization as the ethanol solutions used to prepare the former should have extracted the saponins from the tissues. However, no difference was found between paraffin and cryo-sections, so either saponins have been extracted in the two types of sections (saponins are also soluble in water) or they have not been extracted (possibly because of the fixatives used). In both cases, the use of lectins only cannot give any precise information about the localization of saponins in the Cuvierian tubules.

### Mass spectrometry detection of saponins directly on Cuvierian tubules

**Figure 2:**
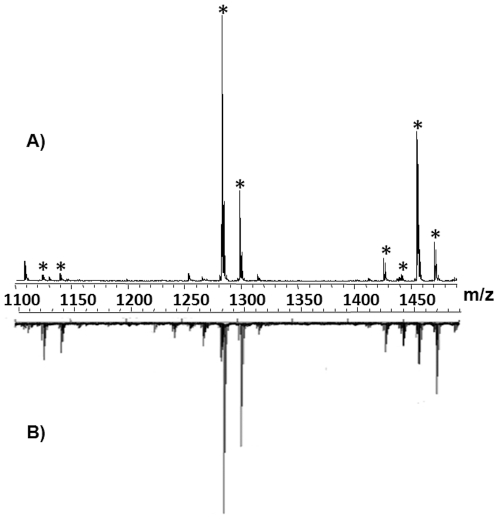
Detection of Saponins by MALDI direct tissue analysis. Comparison of an average mass spectrum of the Cuvierian tubules of *Holothuria forskali* obtained by direct MALDI-MSI (A) and a mass spectrum obtained by classical MALDI-MS analysis after extraction and purification of saponins from the tubules (B). Saponin ions are marked by an asterisk.

After lectin experiments, a direct analysis of a cryo-section through a bundle of a dozen Cuverian tubule was done using a MALDI-Imaging mass spectrometer. Contrary to the usual procedure [29], [30], rinsing steps were not possible due to the high solubility of saponins and the risk of their delocalization or their loss. After matrix deposition, a mass spectrum was averaged by randomly acquired data across the entire tissue section surface (Fig. 2A). This spectrum displays, in the mass range *m/z* 1100 to 1500, eight major peaks at *m/z* 1125, 1141, 1287, 1303, 1433, 1449, 1463 and 1479 (marked with an asterisk in the Figure 2A). In view of their *m/z*, these peaks might correspond to triterpene glycosides. A comparison was done between the MALDI-MS direct tissue profiling spectrum and a mass spectrum from a classical MALDI-tof analysis following chemical extraction of the saponins [17]. The pattern of the spectra was very similar for both types of analyses and presents the same *m/z* ratios with a conservation of the relative abundance of the ions for the eight major peaks (Fig. 2).

**Figure 3:**
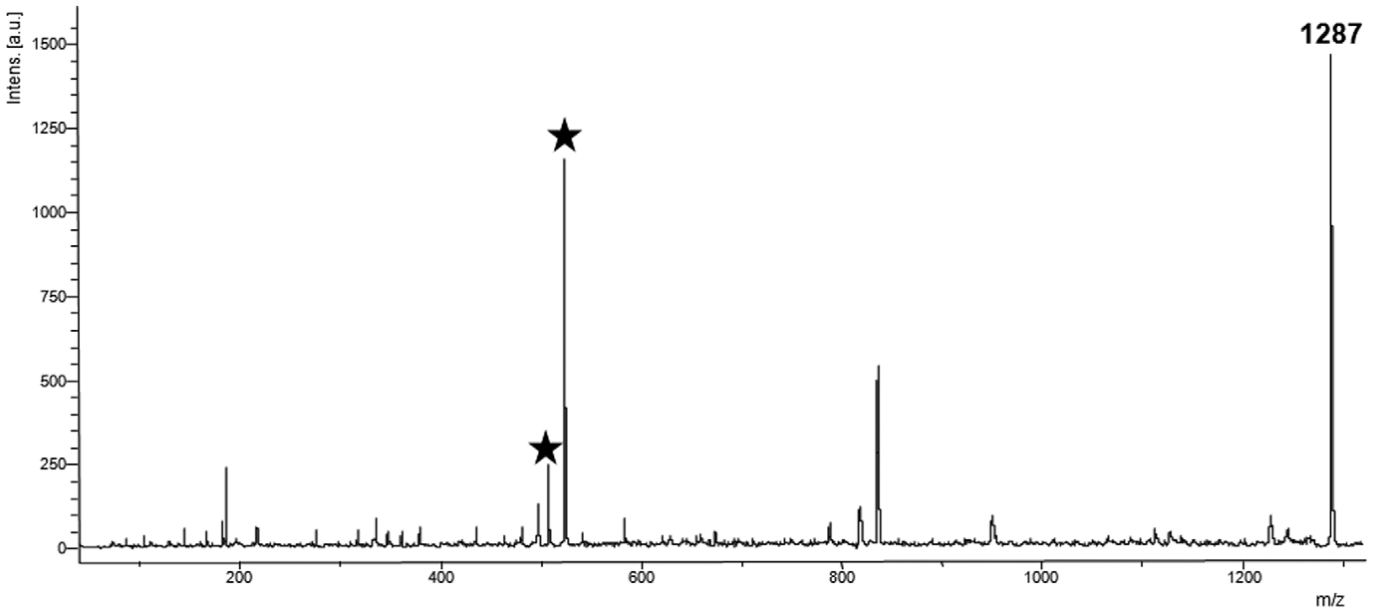
Direct tissue MS/MS analysis of the saponin ions at *m/z* 1287. Characteristic signature peaks of saponins (*m/z* 507 and *m/z* 523) are marked by an asterisk.

**Figure 4:**
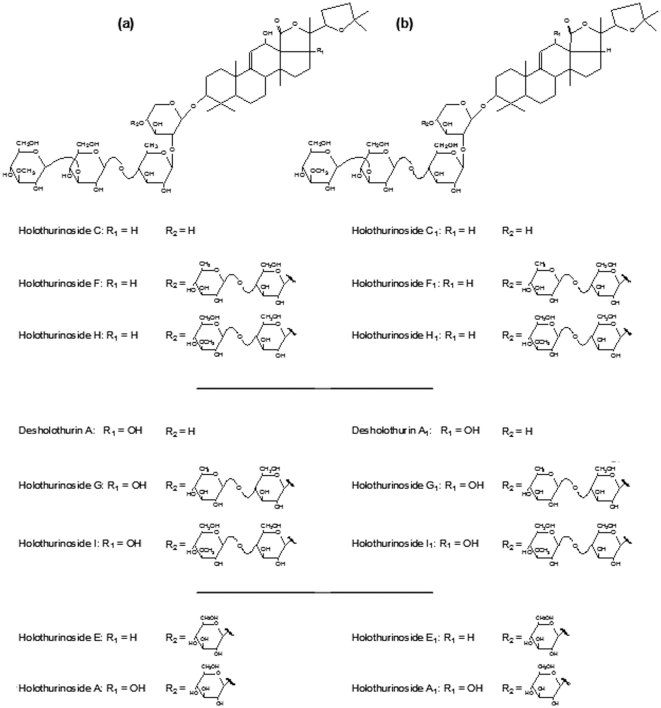
Molecular structures of saponins from the Cuvierian tubules of *Holothuria forskali*. Isomeric congeners are presented in columns (a) and (b). Structures and corresponding names are from Van Dyck *et al.* [17].

In order to obtain more information on the compounds detected by MALDI-MS direct tissue profiling and to confirm that these peaks correspond to saponins, tandem mass spectrometry (MS/MS) was undertaken directly on tissue section. As an example, the MALDI-MS/MS mass spectrum for the ions detected at *m/z* 1287, which present the most intense signal, is depicted in Figure 3. MS/MS spectra of saponins in the Cuvierian tubules were characterized by the presence of two typical signals at *m/z* 507 and 523, corresponding to the oligosaccharidic chains [MeGlc-Glc-Qui +Na^+^] and [MeGlc-Glc-Glc +Na^+^], respectively (Fig. 3). This permitted to highlight the presence of isomers (see [17] for details). In our case, these two ions were observed in the MALDI-MS/MS spectrum realized directly from the tissue section and confirmed that the ions detected at *m/z* 1287 correspond to the isomeric saponins holothurinosides E and E1. Similar fragmentation patterns were obtained for the 8 peaks observed upon MALDI-MS direct profiling of the Cuvierian tubules, each one presenting *m/z* 507 and 523 ions as the key decomposition products. These results confirmed the detection of the 16 principal saponins in Cuvierian tubules (Table 1; Fig. 4). Like in the extraction experiments holothurinosides E and E1 (detected at *m/z* 1287) were characterized by the most intense signal (Fig. 2) and consequently seemed, in first approximation, to be the most abundant saponins in the Cuvierian tubules. On the other hand, holothurinosides C and C1 (at *m/z* 1125) presented the least intense signal.

**Table 1:** Complete list of saponins detected by MALDI Direct Tissue Analysis of a Cuvierian tubule section in *Holothuria forskali*.

[M+Na^**+**^]	Name	Molecular Formula
1125	Holothurinoside CHolothurinoside C_1_	C_54_H_86_O_23_
1141	Desholothurin ADesholothurin A_1_	C_54_H_86_O_24_
1287	Holothurinoside EHolothurinoside E_1_	C_60_H_96_O_28_
1303	Holothurinoside AHolothurinoside A_1_	C_60_H_96_O_29_
1433	Holothurinoside FHolothurinoside F_1_	C_66_H_106_O_32_
1449	Holothurinoside GHolothurinoside G_1_	C_66_H_106_O_33_
1463	Holothurinoside HHolothurinoside H_1_	C_67_H_108_O_33_
1479	Holothurinoside IHolothurinoside I_1_	C_67_H_108_O_34_

This first approach shows that the MALDI-MS direct tissue analysis makes the detection of saponins directly on tissue possible, with results similar to those obtained by conventional techniques, but without any step of extraction and by consuming only a small part of tissue sample.

### Tissue profiling of Cuvierian tubules at different states of stress

**Figure 5:**
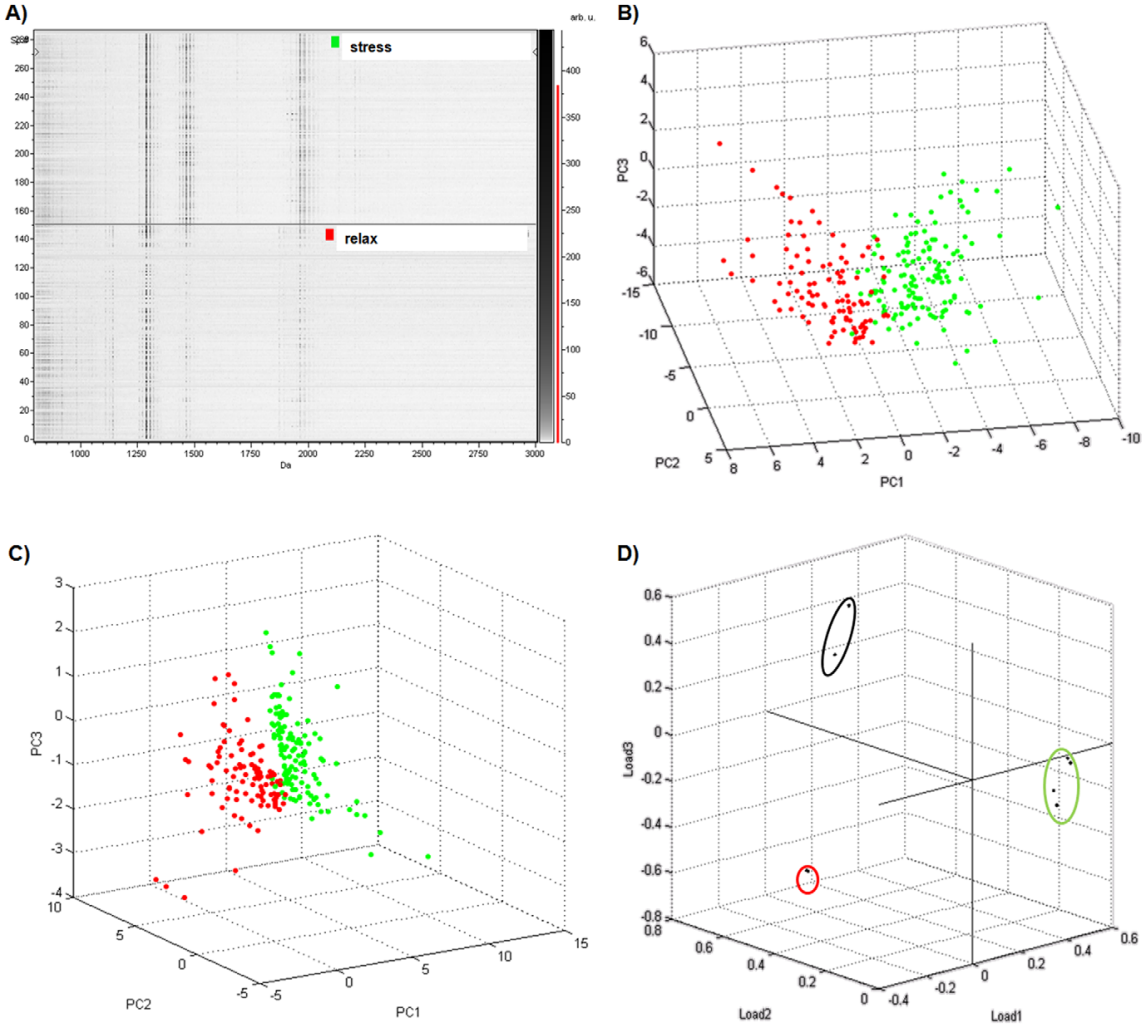
Comparison of saponins composition between stressed and relaxed holothuroids. (A) Pseudogel view of 300 mass spectra from the Cuvierian tubules of *Holothuria forskali*: spectra from stressed holothuroids (upper part) and from relaxed holothuroids (lower part). (B and C) scores plot of first, second and third principal component considering all the ions (B) or only the saponin ions (C) in the MALDI spectra. Each data point represents one mass spectrum (red, relaxed; green, stressed). (D) Scores plot of first, second and third loading considering only the saponin ions. Each data point represents one *m/z* ratio.

The saponin content of Cuvierian tubule sections was investigated by MALDI profiling in order to compare their composition depending on the stress level of the holothuroids. For an in-depth comparison, 3 sections in different parts of a group of tubules were realized for both relaxed and stressed individuals of *H. forskali*. These tissue sections were randomly analyzed by selecting arbitrary positions on the sample. This type of analysis is defined by the fact that the specific spot positions are selected for the data acquisition and that it does not result in a full molecular image. Approximately 150 spots were selected for each condition. For the statistical analyses, the mass spectra were internally recalibrated and normalized on the total ion count. A virtual gel view of individual spectra is presented in Figure 5A. Some differences can be observed from a visual inspection of the spectra, especially in the mass range of saponins. Peaks at *m/z* 1125 and 1141 seemed to be present exclusively in the relaxed state whereas ions at *m/z* 1433, 1449, 1463 and 1479 appeared to be more abundant in a stressed specimen.

To confirm this visual feeling, a statistical analysis was done on the data set. One of the techniques used to reduce the complexity of the information is the PCA [31]. The 3D plots of the first three principal components are shown in Figure 5. When all the ions of the MALDI spectra are considered for PCA analysis, the data were quite scattered (Fig. 5B) but the stressed state data (green dots) were clearly distinguishable from the relaxed state group (red dots). Considering only the saponin peaks for PCA analysis (Fig. 5C), the data were more concentrated in a specific region of the principal component space. Figure 5D showed the result of a 3D loading plot of the first three loadings. Regarding the loading axis, three different groups having a high variance could be observed. The first group was composed of the saponins detected at *m/z* 1125 and 1141 (red oval) and seemed to be more specific to the relaxed state data set. Another group (green oval), comprising peaks at *m/z* 1433, 1449, 1463 and 1479, was characteristic of the stressed state. A third group, between the two others and combining the saponins at *m/z* 1287 and 1303, may also be distinguished. The multivariate analysis used here thus demonstrated the capacity of identifying differential aspects of the profile of ionic species from relaxed or stressed animals. The point to emphasize is that a correct grouping can be achieved by using only the peaks of saponins. Our findings suggest that, in Cuvierian tubules, saponins are the most variable compounds during a stress response.

**Figure 6:**
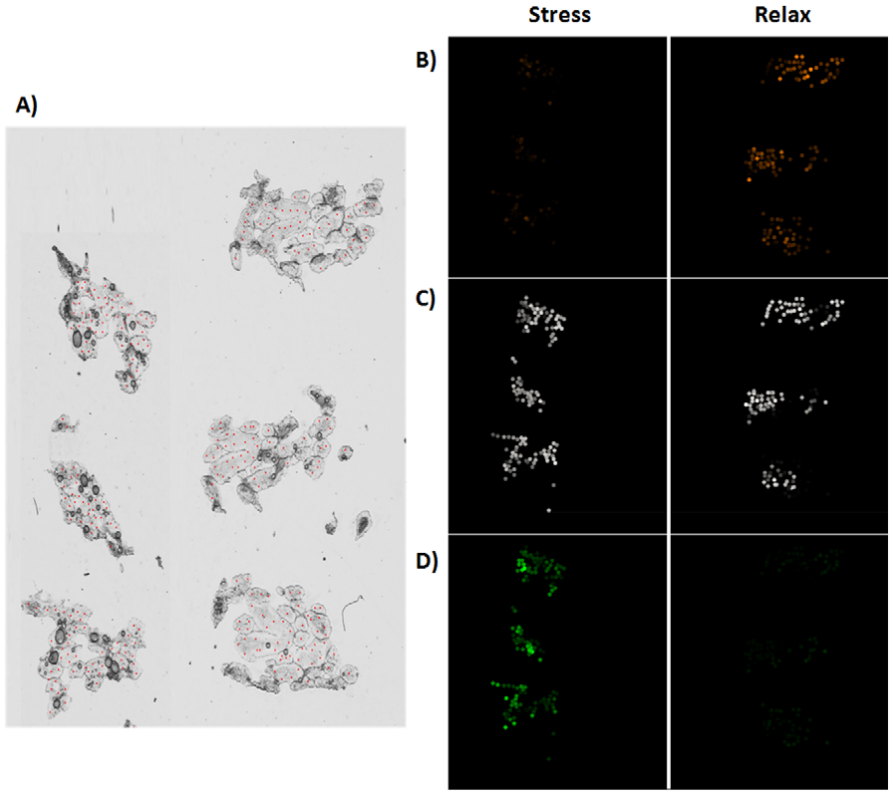
Repartition of saponin groups depending on the state of stress. (A) Optical image of sections through bundles of Cuvierian tubules. The red dots depict the positions at which each mass spectrum was acquired. (B, C and D) Visualization of signal intensity for the group of saponins at *m/z* 1125 and 1141 (B); the group of saponins at *m/z* 1287 and 1303 (C); and the group of saponins at *m/z* 1433, 1449, 1463 and 1479 (D) in tubule frozen sections from stressed (left part) or relaxed (right part) individuals.

With FlexImaging software, it is possible to visualize, using a color gradient, the distribution of the signal obtained for each group of saponins previously defined after PCA on the direct profiling of Cuvierian tubule sections. Results are shown in Figure 6. As expected from the PCA results, the three groups correspond to different physiological states. The group of *m/z* 11xx was more conspicuous in the data of the relaxed state whereas it corresponded to a low signal in the stressed state (Fig. 6B). In comparison, the group of *m/z* 14xx (Fig. 6D) was more specifically correlated to the stressed state. Finally the group of *m/z* 1287 and 1303 (Fig. 6C) was present in the same proportion whatever the physiological state of the animal. In view of these results (summarized in Table 2), some hypotheses can be made concerning the kinetic of saponin use in the Cuvierian tubules during a stress. Holothurinosides C/C1 and desholothurins A/A1 (*m/z* 1125 and 1141) seem to be rapidly used or released outside of the Cuvierian tubules. On contrary, holothurinosides F/F1, G/G1, H/H1 and I/I1 (*m/z* 1433, 1449, 1463 and 1479), appear to be abundantly produced in a state of stress compared to their low level in the relaxed condition. A similar trend has been observed for the saponins present in the body wall of relaxed and stressed individuals of *H. forskali* [32]. In view of the structures of these different saponins, it seems likely that, in case of a prolonged stress situation, holothurinosides C/C1 (*m/z* 1125) are converted to holothurinosides F/F1 and H/H1 (*m/z* 1433 and 1463, respectively), and desholothurins A/A1 (*m/z* 1141) to holothurinosides G/G1 and I/I1 (*m/z* 1449 and 1479, respectively). This is simply done by the addition of a disaccharide: either Qui-Glc or MeGlc-Glc (Fig. 4). These modifications would make the saponins more membranolytic (i.e. more toxic) and more hydrophilic (i.e. more soluble in sea water) [33]. It is not surprising therefore that holothurinoside G (*m/z* 1449) is the only saponin detected in the sea water surrounding relaxed individuals of *H. forskali* [32]. Concerning the last group including the holothurinosides A/A1 and E/E1 (*m/z* 1303 and 1287) their expression level look to be the same in the relaxed or stressed states.

**Table 2:** Abundance of saponins in the Cuvierian tubules of relaxed and stressed individuals of *Holothuria forskali*.

Saponins[M+Na]^**+**^	Relaxed	Stressed
**1125**	**+**	**−**
**1141**	**+**	**−**
**1287**	**+**	**+**
**1303**	**+**	**+**
**1433**	**−**	**+**
**1449**	**−**	**+**
**1463**	**−**	**+**
**1479**	**−**	**+**

The saponins ions are present (+) or absent (−).

### MALDI-Imaging of Cuvierian tubules

MALDI-MS direct tissue analysis is able to detect saponins on Cuvierian tubules and to study the changes occurring during a stress response but our purpose was also to establish if MALDI-MSI could be performed on Cuvierian tubules to visualize the spatial distribution of saponins in the tissues. To achieve a molecular image, a cross section of a dozen of tubules was prepared and a measurement region was defined. Tubules are very small, i.e. a cross section presents a diameter of about 500 µm. To localize the saponins as precisely as possible, the distance between each point was set to 60 µm. In total, a cross section of 15 tubules was analyzed at a spatial resolution of 60×60 µm^2^.

**Figure 7:**
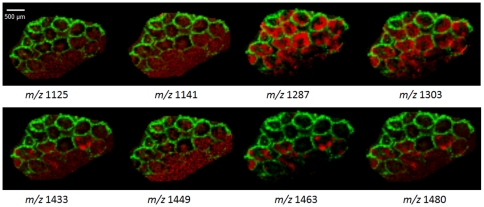
MALDI-Imaging results shown in false colour representation. The color intensity reflects intensities of the selected mass signals: green, ions at *m/z* 843 used as a counterstain; red, the different saponin ions for which the *m/z* ratios are indicated below the images.

Figure 7 shows the molecular images for each *m/z* ratio (corresponding to a pair of isomeric saponins). Ions detected at *m/z* 843 were used as a counterstain due to their specific localization in the mesothelium, thereby defining the tubule outer limit. The data clearly provided evidences that there is a differential distribution of saponins within a cross section of Cuvierian tubules. Ions at *m/z* 1125 and 1141 were slightly detected and without any specific location. This is in agreement with the average spectrum in Figure 2, in which these saponins presented a very low intensity. Concerning ions at *m/z* 1287 and 1303, the most intense in the average spectrum, they had internal localization concentrated in what appears to be the connective tissue. The saponin group at *m/z* 14xx showed a distinct distribution, different from the previous one, except for *m/z* 1449 which presented a more diffuse signal. This group of saponins was only present in some tubules while saponins at *m/z* 1287 and 1303 were distributed in all the tubules imaged. Moreover the distribution was not uniform inside the connective tissue and seemed to concentrate on the outer edges. Thus, in addition to their high abundance irrespective of the physiological state, saponins at *m/z* 1287 and 1303 also show a specific localization in the Cuvierian tubules. It is noteworthy that in the body wall of *H. forskali*, these saponins are very clearly separated spatially from all the other saponins. Indeed, they present a mesothelial or near mesothelial localization (i.e., in the inner body wall epithelium facing the coelomic cavity) while saponins at *m/z* 11xx and 14xx have an epidermal or near epidermal localization (i.e., in the outer body wall epithelium facing sea water) [32].

The fact that saponins at *m/z* 1433, 1463 and 1479 are only present in some tubules could be correlated to the fact that this saponin group would be specific to stressed Cuvierian tubules. They could be specifically expressed in the tubules which are ready to be expelled. Indeed, although individuals of *H. forskali* possess between 200 and 600 tubules, only 10 to 20 of them are fired at a time, presumably in a predefined sequence [27]. Alternatively, the difference of localization of the saponins at *m/z* 14xx could correspond to a mix of fully functional tubules and regenerating tubules. Indeed, to maintain the line of defense, *H. forskali* is capable to regenerate its Cuvierian tubules [28]. During the collection of the tubules directly after dissection, no distinction was possible between complete tubules and regenerating ones.

### Conclusions

We have shown that it is possible to use direct tissue mass spectrometry profiling and MALDI-Imaging to study saponins contained in the Cuvierian tubules of holothuroids. These molecules are soluble in most solvents and their localization is therefore difficult through classical cytochemistry methods. Moreover, saponin detection by histochemistry with lectins is also hampered by their weak specificity towards saponins. In this context, the use of MALDI tissue analysis (profiling or imaging) seemed a good alternative. We have demonstrated that, by using MALDI profiling on Cuvierian tubules, it was possible to detect signals of the 16 principal tubule saponins from *H. forskali*. The comparison obtained by a statistical analysis of data from relaxed and stressed holothuroids indicates that there is a differential composition in saponins depending on the condition of the animal. Finally, we demonstrate that MALDI-Imaging can be used to localize saponins inside Cuvierian tubule sections. An important future extension might be the use of MALDI-Imaging at higher resolution to check if saponins are specific to certain cell types. Indeed, the current resolution is too low for this purpose. However, comparing the saponin-specific MALDI-MSI images with the high-resolution but non-specific lectin-labeled images suggests that saponins would be produced at the level of the connective tissue layer. Holothurinosides A/A1 and E/E1 (*m/z* 1303 and 1287, respectively) could originate from the vacuolar cells, while the other congeners would be produced by the neurosecretory-like cells. All the results taken together indicate a complex chemical defense mechanism with, for a single organ, different sets of saponins originating from different cell populations and presenting different responses to stress.

Finally, this study reflects that MALDI profiling and MALDI-MSI are valuable tools for chemical ecology studies in which specific chemical signaling molecules like allelochemicals or pheromones have to be tracked [34], [35]. This report represents one of the very first studies using these tools to provide a functional and ecological understanding of the role of natural products from marine invertebrates.

## Methods

### Chemicals and materials

α-cyano-4-hydroxycinnamicacid (HCCA), trifluoroaceticacid (TFA), acetonitrile (ACN), aniline (ANI), were purchased from Sigma-Aldrich (Saint-Quentin Fallavier, France).

### Sampling

Individuals of *H. forskali* were collected at depths ranging from 10 to 30 m by scuba diving at the Biological Station of Banyuls-sur-Mer (Pyrénées-Orientales, France). They were transported to the Marine Biology Laboratory of the University of Mons, where they were kept in a marine aquarium with closed circulation (13°C, 33‰ salinity). Two different physiological states were investigated: relaxed and stressed individuals (only the former were used, however, for the histochemical approach). Relaxed animals were anesthetized 2 h in a 0.1% solution of 1-phenoxy-2-propanol (Sigma-Aldrich, Germany) in sea water. Stressed individuals were mechanically disturbed for 4 h by repetitive hitting using a specific device. The device consisted of three loads hanging from a horizontal rotating bar and immerged in the tank containing the animal. Loads were embedded in parafilm in order to avoid injuries to the body wall. Animals stimulated in this way contracted but never expelled their Cuvierian tubules. After each treatment, the holothuroids were dissected in order to collect their Cuvierian tubules. A fraction of the tubules was stored in 70% ethanol for saponin extraction, another fraction was fixed for histology and histochemistry, and a last fraction was quickly frozen in liquid nitrogen for MALDI-MSI.

### Lectin assays

Lectins are proteins or glycoproteins of non-immune origin that are able to bind carbohydrates without chemically modifying them [36]. Three glucose- and mannose-specific, biotinylated lectins purchased from Vector Laboratories (Burlingame, CA) were tested on saponin extracts from Cuvierian tubules: concanavaline A (Con A), *Lens culinaris* agglutinin (LCA) and *Pisum sativum* agglutinin (PSA).

Saponins were extracted as described in [17]. Briefly, the homogenized tissue was extracted twice with ethanol:water (70∶30) followed by filtration. The extract was then evaporated at low pressure in a double boiler at 30°C using a rotary evaporator (Laborota 4001 efficient, Heidolph). The dry extract was diluted in 90% methanol and partitioned against n-hexane (v/v). The water content of the hydromethanolic phase was adjusted to 20% (v/v) then to 40% (v/v), these solutions being partitioned against CH_2_Cl_2_ and CHCl_3_, respectively. Finally, the hydromethanolic solution was evaporated and dissolved in water in order to undergo chromatographic purification. The crude aqueous extract was placed on a column of Amberlite XAD-4 (Sigma-Aldrich, St. Louis, MO). Washing the column with water removed the inorganic salts and subsequent elution with methanol allowed to recover saponins. The methanolic phase was then evaporated and the dry extract was diluted in water in order to undergo a last partitioning against iso-butanol (v/v). The butanolic fraction contained the purified saponins.

The final butanol fraction was spotted onto strips from two different supports: nitrocellulose membranes (BA 85, Schleicher & Schuell, Dassel, Germany) and thin layer chromatography plates (TLC plates, silica gel on aluminium, Sigma-Aldrich, St. Louis, MO). A diluted aqueous solution of commercial milk powder was used as a positive control. The strips were pre-incubated for 30 min in phosphate-buffered saline containing 0.05% of Tween 20 (MP Biomedicals, Solon, OH) and 0.1% bovine serum albumin (Sigma, St. Louis, MO) (PBS-Tween-BSA), rinsed in PBS, and were then incubated for 2 h at room temperature in the biotinylated lectins diluted at 0.25 µg/ml in PBS-Tween-BSA. After several washes in PBS, the strips were incubated for 1 h in a solution of streptavidine-conjugated alkaline phosphatase (Vector Laboratories, Burlingame, CA) diluted 1∶500 in PBS-Tween-BSA, then washed again in PBS-Tween-BSA. Finally, the strips were incubated for 10 min in a 2% solution of NBT/BCIP (Roche, Mannheim, Germany) in the revelation buffer (0.1 M Tris-HCl, pH 9.5, 0.1 M NaCl, 0.05 M MgCl_2_). The reaction was stopped in deionized water.

### Histology and histochemistry

Cuvierian tubules were fixed in Bouin's fluid, in 10% formalin in sea water, or in 4% paraformaldehyde in PBS. Bouin's fluid-fixed tubules were dehydrated in graded ethanol, embedded in paraffin wax using a routine method, and sectioned at 7 µm using a rotary microtome (Microm HM340E). Formalin- and paraformaldehyde-fixed tubules were embedded in a commercial tissue freezing medium (TBS from Triangle Biomedical Sciences, Durham, N.C.) in order to realize 10 µm cryo-sections using a Leica Cryocut 1800 cryostat operated at −25°C. Some sections (paraffin- and cryo-sections) were stained with Heidenhain's azan trichrome stain, with Masson's trichrome stain or with the periodic acid-Schiff (PAS) method. Other sections were subjected to an indirect histochemical labeling method.

For lectin histochemistry, non-specific background staining was blocked by a 30 min pre-incubation of the sections in PBS containing 0.05% of Tween 20 and 3% BSA (PBS-Tween-BSA2). Biotinylated lectins, diluted at 2.5 or 0.25 µg/ml in PBS-Tween-BSA2, were then applied for 2 h at room temperature. After several washes in PBS, the sections were incubated for 30 min in the ABComplex solution (Dako, Denmark) consisting of streptavidin and biotinylated peroxidase. Following a final wash in PBS, lectin-carbohydrate complexes were revealed by incubation of the sections with a 0.05% solution of DAB (3,3′-diaminobenzidine tetrahydrochloride; Sigma, St. Louis, MO) in PBS containing 0.02% H_2_O_2_. The reaction was controlled under microscope and, when the optimal contrast was obtained, the revelation was stopped in deionized water. Finally, the sections were lightly counterstained with hemalum and luxol blue and observed with a Zeiss Axioscope A1 microscope (Carl Zeiss MicroImaging, Göttingen, Germany) equipped with a Zeiss Icc-3 digital camera.

### Tissue preparation for mass spectrometry

Thin 12 µm tissue sections were obtained from a frozen bundle of Cuvierian tubules using a cryostat CM1510S (Leica Microsystems, Nanterre, France) and applied onto an indium-tin oxide (ITO)-coated conductive glass slides (Bruker Daltonics, Bremen, Germany).

A solid ionic HCCA/ANI matrix was used to analyze the saponins on tissue sections and was prepared following a previously established procedure [37]. Briefly 7.2 µl of ANI (1.5 equivalent) was added to a solution containing 10 mg/ml of HCCA (1 equivalent) in ACN/aqueous TFA 0.1% (6∶4, v/v). The matrix solution was then deposited using an automatic vibration vaporization system (ImagePrep, Bruker Daltonics, Bremen, Germany) to cover the whole surface of the tissue section. The ImagePrep method for HCCA/ANI deposition is based on a modified HCCA method included in the ImagePrep.

### MALDI-MS direct tissue analysis

Data were acquired at different positions on the tissue section using an UltraFlex II MALDI-TOF/TOF mass spectrometer (Bruker Daltonics, Bremen, Germany) equipped with a Smartbeam ™ laser. Direct profiling data were performed in positive reflectron mode, for a mass range from *m/z* 800 to 3000. A total of 300 spectra were acquired at each spot at a laser frequency of 100 Hz. FlexImaging ™ 2.1 software was used for automatic data acquisition of 150 spots for each condition. Standards for spectral calibration consisted of a mixed solution of peptides ranging between *m/z* 900 to 3500 Da.

### PCA statistical analysis

Statistical analyses were performed using ClinProTools 2.2 software (Bruker Daltonics, Bremen, Germany). After loading data of each condition, mass spectra were internally recalibrated and normalized. An average spectrum created from all single spectra was used for peak picking. A first PCA analysis was done using all peaks detected in the average mass spectrum. A second PCA was completed using only peaks corresponding to the saponins.

### MALDI-Imaging

The images were acquired using an UltraFlex II MALDI-TOF/TOF mass spectrometer (Bruker Daltonics, Bremen, Germany) equipped with a Smartbeam™ laser. Images data were performed in positive reflectron mode, and MALDI-MS spectra were acquired in the *m/z* range from *m/z* 800 to 3000. A total of 300 spectra were acquired at each spot at a laser frequency of 100 Hz. Spatial resolution was set to 60×60 µm^2^. FlexImaging ™ 2.1 software was used for molecular image reconstruction. Standards for spectral calibration consisted of a mixed solution of peptides ranging between *m/z* 900 to 3500 Da.
